# Impact of Marginalization Dimensions on Survival Disparities in Epithelial Ovarian Cancer: An Ontario Population-Based Study

**DOI:** 10.3390/cancers18121892

**Published:** 2026-06-10

**Authors:** Justin Wei-Jia Lim, Lilian T. Gien, Zharmaine Ante, Ning Liu, Lauren Philp, Keerat Grewal, Genevieve Bouchard-Fortier

**Affiliations:** 1 Department of Obstetrics and Gynecology, University of Toronto, Toronto, ON M5G 1E2, Canada; justinwj.lim@mail.utoronto.ca (J.W.-J.L.);; 2 Division of Gynecologic Oncology, Sunnybrook Health Sciences Centre, Toronto, ON M4N 3M5, Canada; 3 ICES Central, Toronto, ON M4N 3M5, Canada; 4 Institute of Health Policy, Management and Evaluation, Toronto, ON M5T 3M6, Canada; 5 Division of Gynecologic Oncology, Princess Margaret Cancer Centre, University Health Network, Sinai Health System, Toronto, ON M5G 2M9, Canada; 6 Division of Emergency Medicine, Department of Medicine, University of Toronto, Toronto, ON M5G 1V7, Canada; 7 Schwartz/Reisman Emergency Medicine Institute, Sinai Health, Toronto, ON M5G 1X5, Canada

**Keywords:** ovarian cancer, survival, social determinants of health, marginalization, health equity

## Abstract

**Objectives:** We aimed to examine associations between social marginalization, defined by the Ontario Marginalization Index (“ON-Marg”), and overall survival (OS) in epithelial ovarian cancer (EOC). **Methods**: This was a population-based retrospective cohort study using linked administrative data in Ontario, Canada, including adults ≥ 18 years diagnosed with stage II-IV EOC (2010–2022). ON-Marg dimensions included Material Resources (economic disadvantage), Households and Dwellings (housing type/density), Age and Labour Force (workforce participation), and Racialized and Newcomer Populations (recent immigrants/visible minorities), and were categorized into quintiles (Q1 least marginalized, Q5 most marginalized). The primary outcome was OS. Multivariable Cox models estimated adjusted hazard ratios (aHR) for each ON-Marg dimension. Wald χ^2^ statistics identified the dimension most strongly associated with OS. **Results**: Material Resources was most strongly associated with OS. Compared with Q1 (least marginalized), higher mortality was observed in Q3 (aHR 1.10; 95%CI 1.02–1.19), Q4 (aHR 1.13, 95%CI 1.05–1.22), and Q5 (aHR 1.25, 95%CI 1.15–1.35). Greater marginalization in the Racialized and Newcomer Populations dimension was associated with improved OS (Q5 aHR 0.87, 95%CI 0.80–0.94). The association between Material Resources and OS persisted in patients undergoing cytoreductive surgery with chemotherapy, but not among those receiving chemotherapy alone or no treatment. **Conclusions**: Material Resources is an independent predictor of survival in EOC within a universal, publicly funded healthcare system, with greatest impact among patients undergoing multimodal oncologic care. Residence in highly racialized or newcomer communities was associated with improved survival. Material marginalization is highlighted as a key driver of inequity, supporting targeted system-level interventions to address financial and logistical barriers to care.

## Simple Summary

This Canadian population-based study of 9613 patients with stage II–IV epithelial ovarian cancer examined how different dimensions of social marginalization, measured by the Ontario Marginalization Index, influence overall survival within a universal healthcare system. Material deprivation emerged as the strongest predictor of worse survival, with the most materially deprived patients having a 25% higher hazard of death compared to the least deprived, even after adjusting for age, stage, comorbidity, and treatment. This survival gradient was most pronounced among patients undergoing complex, multimodal treatment (primary cytoreductive surgery or neoadjuvant chemotherapy with interval debulking surgery), suggesting that material barriers disproportionately affect patients navigating prolonged treatment pathways. In contrast, residence in neighbourhoods with higher racialized and newcomer population composition was associated with improved survival. These findings demonstrate that universal health insurance alone does not eliminate survival disparities driven by material deprivation and support the need for targeted system-level interventions addressing financial and logistical barriers to complex oncologic care.

## 1. Introduction

Epithelial ovarian cancer (EOC) is the leading cause of death among gynecologic malignancies, with five-year overall survival (OS) below 50% despite advances in cytoreductive surgery and systemic therapy [1]. While prognosis is traditionally attributed to tumour biology, stage, and treatment-related factors [2], substantial variation in survival persists even after accounting for these factors, suggesting influences beyond conventional prognostic indicators [3]. These variations in survival outcomes can be seen in Canada, despite a strong public healthcare system which provides universal access to medically necessary care [4]. Growing evidence across multiple cancer sites suggests that social inequalities contribute to oncologic outcomes and may account for these survival disparities [5,6,7,8,9].

Social determinants of health (SDH) encompass the non-medical context into which individuals are born, live, work, and age through, and can impact both health status and engagement with the healthcare system [10]. Inequities driven by a complex interplay of socioeconomic, geographic, political, and historical factors have resulted in disparities in care for underserved populations in Canada [5]. These contextual factors can shape one’s access to care along the entire cancer care continuum [5,11,12]. This in turn could influence an individual’s survival from EOC via multiple mechanisms, including a delay in healthcare presentation, differences in rates of guideline-concordant treatments, or variability in adherence to surveillance recommendations [13,14,15]. Emerging evidence suggests SDH, such as socioeconomic status, education, household characteristics, minority status, and housing circumstances, may influence EOC survival independent of established clinical factors [13,16,17].

Most research on health disparities in cancer has focused on malignancies with well-defined behavioural risk factors or established screening programmes, where causal links between social marginalization and worsened outcomes are more apparent. In contrast, EOC lacks clear social risk factors and does not benefit from screening, making disparities related to marginalization potentially underexplored. Studies from the United States (US) have shown that income, housing, and race influence EOC survival, largely through differences in access to and quality of care [13,16,17]. Although individual risk factors such as patient demographics, cancer biology, and treatment strategies are comparable between the US and Canada, the Canadian healthcare system is quite distinct due to its universal, publicly funded structure. Historical, political, and cultural contexts differ substantially between the two countries, further shaping how SDH may affect clinical outcomes, making studies originating from the US challenging to interpret in a non-American context. To date, there are limited studies that have evaluated the impact of marginalization on the survival of EOC patients in Canada. Given this gap, the primary objective of this study was to evaluate the impact of different marginalization dimensions on OS of EOC patients in Ontario, Canada and to identify dimensions of marginalization that most strongly influence survival outcomes to inform targeted areas of future work.

## 2. Materials and Methods

### 2.1. Data Sources

We conducted a population-based retrospective cohort study of patients with stage II to IV EOC using linked administrative databases housed at ICES (formerly known as the Institute for Clinical Evaluative Sciences), an independent, non-profit research institute whose legal status under Ontario’s health information privacy law allows it to collect and analyze health care and demographic data, without consent, for health system evaluation and improvement. The use of the data in this project is authorized under Section 45 of Ontario’s Personal Health Information Protection Act (PHIPA) and does not require review by a Research Ethics Board. As all residents of Ontario (population 15.5 million) are universally insured under the publicly funded Ontario Health Insurance Plan (OHIP) for medically necessary hospital and physician services, these data are comprehensive. Special populations whose healthcare is federally funded are excluded from Ontario’s provincial health administrative databases, including refugee claimants, incarcerated people, Canadian Armed Forces, and Indigenous people living on reserves.

Datasets were linked using unique encoded identifiers and analyzed at ICES: the Ontario Cancer Registry (OCR), which captures all incident cancers in Ontario; the Ontario Marginalization Index (ON-Marg), which contains census-derived neighbourhood indictors of marginalizations across multiple dimensions; the Registered Persons Database (RPDB), containing patient demographics and vital status; The Canadian Institute for Health Information (CIHI) Discharge Abstract Database (CIHI-DAD), Same Day Surgery Database (CIHI-SDS), and National Ambulatory Care Reporting System (NACRS), containing diagnostic and procedural information for all inpatient, outpatient, and emergency department encounters; and the OHIP database, capturing physician billing claims for health services.

### 2.2. Study Population

**Figure 1:**
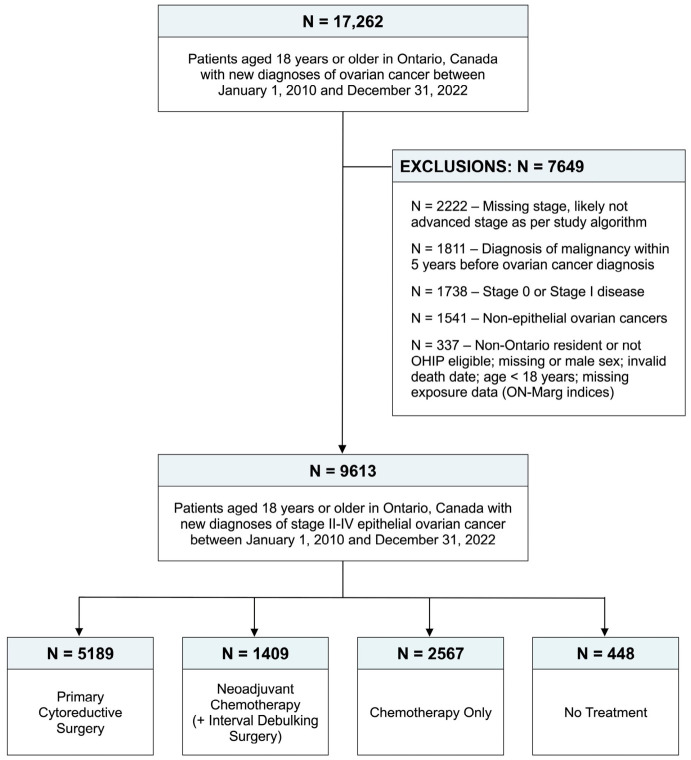
Population-Based Retrospective Cohort: New diagnoses of stage II to IV epithelial ovarian cancer in patients 18 years or older in Ontario, Canada, between 1 January 2010 and 31 December 2022.

As outlined in Figure 1, the cohort included all adults (≥18 years) diagnosed with stage II to IV EOC between 1 January 2010 and 31 December 2022 in Ontario, Canada. Eligible ovarian cancer patients were identified in the OCR database using the International Classification of Disease for Oncology, 3rd Edition, topography codes (C48, C56, C57). Patients were excluded if they (1) had non-epithelial histology, (2) had stage I disease, (3) had a prior malignancy within five years prior to EOC diagnosis, (4) were missing ON-Marg indices, or (5) were not OHIP eligible at diagnosis date. Stage I EOC was excluded, as stage II-IV disease represents more than 80% of diagnoses and involves complex, multimodal treatment pathways where marginalization factors are more likely to impact outcomes. Patients were then followed from their index date of diagnosis up to 31 December 2024, allowing for at least two years of follow-up for all patients.

For patients with missing stage information, we used a previously developed algorithm to classify patients as likely having advanced-stage disease at presentation (“Unknown, Advanced”) based on patterns of treatment and surgical codes within defined time windows relative to the date of diagnosis [18]. Specifically, patients with missing stage were classified as advanced-stage if they met any of the following criteria: (1) receipt of chemotherapy within 6 months prior to their first gynecologic or multivisceral cytoreductive surgery; (2) receipt of a multivisceral cytoreductive surgery (e.g., bowel resection) from 30 days before to 12 months after diagnosis, regardless of the timing of gynecologic surgery; (3) receipt of chemotherapy from 30 days before to 6 months after diagnosis in the absence of any surgery; or (4) receipt of neither surgery nor chemotherapy within the specified time windows.

### 2.3. Exposure

The primary exposure of marginalization was defined using the ON-Marg index, an area-level measure developed by the Centre for Urban Health Solutions at St. Michael’s Hospital utilizing data from the Canadian Census [19]. ON-Marg uses census-derived indictors to quantify four distinct dimensions of marginalization across census tracts and dissemination areas (smallest census geographic unit linked to postal codes). The four dimensions of marginalization defined by ON-Marg include: (1) Material Resources, (2) Households and Dwellings, (3) Age and Labour Force, and (4) Racialized and Newcomer Populations.

Material Resources reflects economic disadvantage, including indicators of access to basic material needs such as the percentage of individuals living on low income, those who are unemployed, and the prevalence of lone-parent families [19]. Households and Dwellings reflects aspects of housing and mobility, including indicators of types and density of accommodations and family structure, such as the proportion of people living alone or renting [19]. Age and Labour Force reflects population age structure and workforce participation, including indicators such as the proportion of seniors (aged 65 years or older) and individuals not engaged in the labour force [19]. Racialized and Newcomer Populations reflect social and cultural factors, including the percentage of recent legal immigrants (arriving within the past five years) and those who identify as visible minorities [19]. During our study period, Canada used a points-based immigration system by assigning points for desirable factors such as education, age, language skills, work experience and job offers.

Within our accrual dates, ON-Marg indices are available for census years (2011, 2016, and 2021). For each dimension, ON-Marg is reported in quintiles (Q), with Q1 representing the least marginalized and Q5 the most marginalized individuals. The ON-Marg score for each dimension of marginalization closest to the patient’s index diagnosis date was utilized. Consistency across iterations has been previously demonstrated, with 81–96% of geographic areas showing limited or no changes in quintile assignment depending on the ON-Marg dimension [20].

### 2.4. Outcomes and Covariates 

The primary outcome was OS, defined as time from diagnosis to death from any cause, with censoring at loss of OHIP eligibility or end of follow-up (31 December 2024). Progression-free survival could not be determined as recurrence data is not captured in the administrative databases.

Patient characteristics were chosen a priori and included age at diagnosis, year of diagnosis, stage at diagnosis (The American Joint Committee on Cancer [AJCC] staging system), type of initial treatment (primary cytoreductive surgery [PCS], neoadjuvant chemotherapy with interval debulking surgery [NACT], chemotherapy only, or no treatment), and level of pre-existing comorbidity. The Elixhauser Comorbidity Index was used to quantify comorbidities by categorizing 31 pre-defined comorbidities of patients to predict hospital resource use and inpatient mortality [21]. For our study, comorbidity scores were calculated and classified as 0–3 or ≥4.

Treatment groups were classified using procedural, surgical, and billing codes from CIHI-DAD, CIHI-SDS, and OHIP databases. Gynecologic or multi-visceral cytoreductive surgeries were attributed to EOC diagnoses if occurred within thirty days prior to diagnosis to 1 year after diagnosis. The PCS group was defined as those who did not have chemotherapy within the six months prior to their first identified surgery, while the NACT group was defined as those that did have chemotherapy within the six months prior to their first identified surgery. For patients without identified surgeries, their primary treatment was defined as chemotherapy alone if they received chemotherapy between the thirty days prior to diagnosis to six months after diagnosis. All remaining patients were considered to have no treatment.

All variables used in the analysis had complete data for included patients, as patients with missing ON-Marg indices or OHIP eligibility were excluded at cohort entry. Stage missingness (36% of the final cohort) was addressed through the validated algorithm described above.

### 2.5. Statistical Analysis

Baseline characteristics were summarized using descriptive statistics. For each ON-Marg dimension, unadjusted overall survival across ON-Marg quintiles was examined using Kaplan–Meier (KM) curves and log-rank tests. Four separate multivariable Cox proportional hazard models were constructed, each including one marginalization domain and adjusting for the same covariates (age-, year-, and stage at diagnosis, Elixhauser comorbidity index, initial treatment). Hazard ratios (HR) with 95% confidence intervals were estimated for each quintile compared to the least marginalized quintile using Cox proportional hazard models. The proportional hazards assumption was assessed for each model using cumulative martingale residual-based supremum tests with resampling simulation methods. To identify the dimension most strongly associated with OS, Type III Wald χ^2^ statistics and model fit criteria (AIC, −2 Log Likelihood) were compared across the four models. Subgroup analyses were then performed for this dimension, stratifying by treatment groups, to evaluate associations within treatment strata rather than compare survival across treatment groups. To evaluate the potential impact of patients with inferred advanced-stage disease, sensitivity analyses were performed excluding all patients with missing stage classified as “Unknown, Advanced” by the staging algorithm. All analyses were conducted at ICES using SAS Enterprise Guide Version 9.4 (SAS Institute Inc., Cary, NC, USA).

## 3. Results

### 3.1. Cohort Characteristics

Of 17,262 individuals diagnosed with ovarian cancer in Ontario between 2010 and 2022, 9613 were included in the final cohort (Figure 1). The mean age was 64 ± 13 years with a mean follow-up of 3.84 ± 3.41 years. Among the final cohort, stage at presentation included 783 (8%) with stage II disease, 3671 (38%) stage III, 1705 stage IV (18%), and 3454 (36%) having missing but advanced stage as identified by the algorithm. Initial treatment included PCS in 5189 (54%) patients, NACT in 2567 (27%) patients, chemotherapy alone in 1409 (15%) patients, and no treatment for 448 (5%) patients. Additional demographics are shown in Table 1.

### 3.2. Unadjusted Survival Analyses

**Figure 2:**
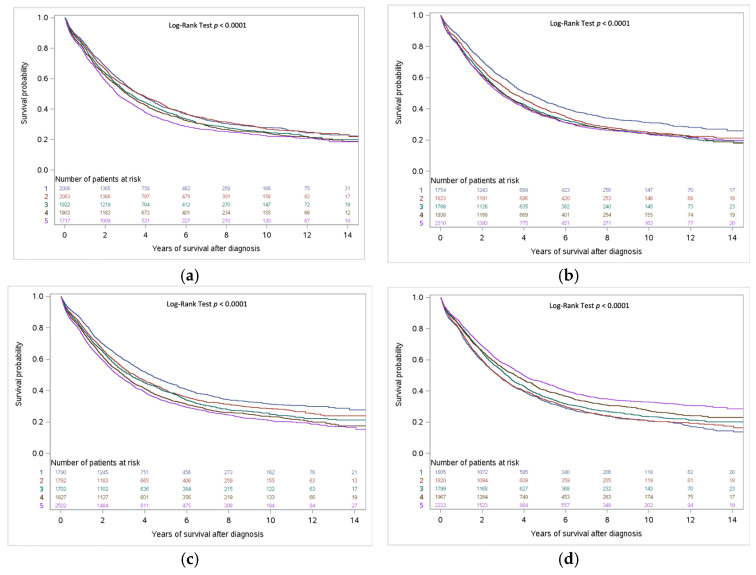
Unadjusted Kaplan–Meier curves demonstrating overall survival for each ON-Marg dimension per quintile: (**a**) Material Resources; (**b**) Households and Dwellings; (**c**) Age and Labour Force; (**d**) Racialized and Newcomer Populations.

Overall survival differed significantly across marginalization quintiles for all ON-Marg dimensions on univariate analysis (Figure 2; log-rank *p* < 0.0001 for each). For Material Resources, median OS declined with increasing marginalization quintiles, decreasing from 3.68 years in Q1 to 2.60 years in Q5. A similar pattern was observed for Households and Dwellings, with median OS decreasing from 4.13 years in Q1 to 2.85 years in Q5. For Age and Labour Force, median OS also declined from 4.28 years in Q1 to 2.72 years in Q5. In contrast, for Racialized and Newcomer Populations, median OS increased with higher quintiles of marginalization (2.79 years in Q1 to 4.02 years in Q5).

### 3.3. Adjusted Survival Analyses

In multivariable models, no significant violations were detected for the primary exposure variables in the Material Resources, Households and Dwellings, or Age and Labour Force models. In the Racialized and Newcomer Populations model, the Q3 quintile demonstrated evidence of nonproportionality (*p* = 0.002), whereas the remaining quintiles did not. Given that this isolated violation was limited to a single intermediate category without a consistent pattern across quintiles, the Cox model was retained without time-varying modification.

**Figure 3:**
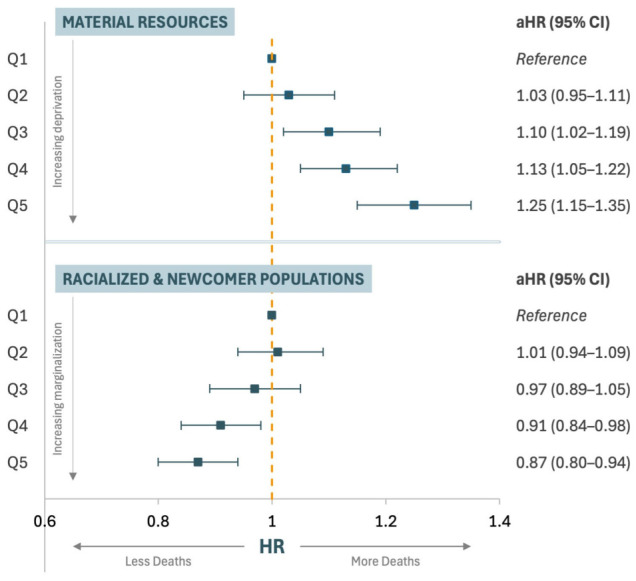
Adjusted hazard ratios and 95% CIs for overall survival from two independent Cox proportional hazards models (for ON-Marg dimensions including Material Resources and Racialized and Newcomer Populations), each modelled separately, adjusted for the same covariates including age at diagnosis, year at diagnosis, stage at diagnosis, initial treatment received, and Elixhauser comorbidity index.

Across the four multivariable Cox regression models (Table 2, Figure 3), the ON-Marg dimension that was most strongly associated with overall survival was Material Resources (Type III Wald χ^2^ 37.29). The Material Resources model showed a gradient association across quintiles, with more marginalized quintiles being associated with increasingly elevated hazards of death when compared to the least marginalized quintile (Q1). While Q2 was not significantly different from this reference (aHR 1.03, 95% CI 0.95–1.11), all other more marginalized quintiles (Q3–Q5) had significantly higher aHRs including Q3 (aHR 1.10, 95% CI 1.02–1.19), Q4 (aHR 1.13, 95% CI 1.0–1.22), and Q5 (aHR 1.25, 95% CI 1.15–1.35).

For Households and Dwellings, all marginalized quintiles (Q2–Q5) had statistically significant associations when compared to the least marginalized quintile (Q1), but effect sizes were small and inconsistent (aHR ranging from 1.09 to 1.13). For Age and Labour Force, most quintiles did not reach statistical significance, and no clear directional pattern was observed. The Racialized and Newcomer Populations model demonstrated lower hazards of death with increasing marginalization, with Q4 (aHR 0.91, 95% CI 0.84–0.98) and Q5 (aHR 0.87, 95% CI 0.80–0.94) demonstrating lower hazards for death.

### 3.4. Treatment Subgroup Analysis—Material Resources

**Figure 4:**
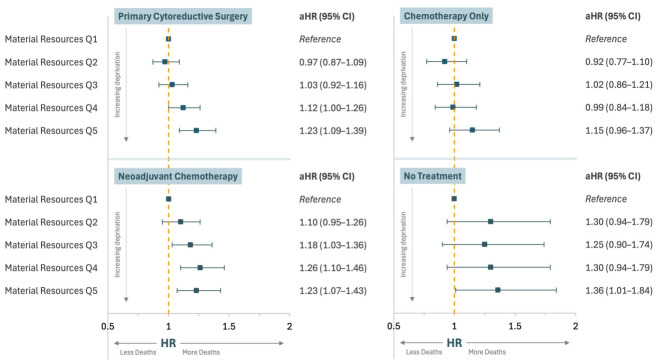
Adjusted hazard ratios and 95% CIs for overall survival from Material Resources Cox proportional hazards model, stratified by initial treatment type (PCS—primary cytoreductive surgery; NACT—neoadjuvant chemotherapy followed by interval debulking surgery; chemotherapy only; no treatment). Models additionally adjusted for age at diagnosis, year at diagnosis, stage at diagnosis, and Elixhauser comorbidity index.

As Material Resources demonstrated the strongest association with OS in the primary analysis, we conducted subgroup analyses for this dimension stratified by treatment (Figure 4). Baseline unadjusted median OS was 70.7 months for PCS, 36.0 months for NACT, 12.4 months for chemotherapy only and 2.3 months for no treatment. These treatment-stratified analyses were designed to evaluate associations between marginalization and OS within treatment strata, rather than to compare survival across treatment groups. Associations between marginalization quintile and OS were observed for patients treated with surgery and chemotherapy (PCS or NACT), whereas no consistent associations were seen for non-surgical patients (chemotherapy only or no treatment). Among patients who underwent PCS, aHR for death rose with increasing marginalization in the Material Resources dimension with more marginalized quintiles demonstrating significantly increased hazards of death (Q4: aHR 1.12, 95% CI 1.00–1.26; and Q5: aHR 1.23, 95% CI 1.09–1.39). Similar findings were noted for patients who received NACT (Q3: aHR 1.18, 95% CI 1.03–1.36; Q4: aHR 1.26, 95% CI 1.10–1.46; and Q5: aHR 1.23, 95% CI 1.07–1.43). In the chemotherapy only subgroup, none of the quintiles differed significantly from the reference. Among patients who received no treatment, estimates were imprecise with wide confidence intervals. In this group, only the most marginalized quintile (Q5) had a significant association with death (aHR 1.36, 95% CI 1.01–1.84).

### 3.5. Sensitivity Analysis

Sensitivity analyses were performed excluding all 3454 patients (35.9%) with inferred advanced stage, restricting the cohort to 6159 patients with known stage II–IV disease. Baseline characteristics stratified by stage category are presented in Supplementary Table S1.

In multivariable Cox models restricted to patients with known stage (Supplementary Table S2), Material Resources remained the dimension most strongly associated with overall survival, with increasing hazards observed across higher quintiles (Q2: aHR 1.01, 95% CI 0.92–1.11; Q3: aHR 1.08, 95% CI 0.98–1.18; Q4: aHR 1.11, 95% CI 1.01–1.22; and Q5: aHR 1.21, 95% CI 1.10–1.33). The Households and Dwellings dimension demonstrated associations with worse survival across higher quintiles (Q5: aHR 1.14, 95% CI 1.03–1.25), while the Age and Labour Force dimension showed no statistically significant associations. The protective association for the Racialized and Newcomer Populations dimension persisted (Q4: aHR 0.89, 95% CI 0.81–0.98; and Q5: aHR 0.89, 95% CI 0.81–0.98).

Treatment-stratified analyses for Material Resources excluding inferred-stage patients (Supplementary Table S3) were also consistent with the primary analysis. Among patients who underwent PCS (*n* = 3236), Q5 was associated with a significantly elevated hazard of death (aHR 1.16, 95% CI 1.01–1.34). Among patients who received NACT (*n* = 1708), Q5 was similarly associated with worse survival (aHR 1.19, 95% CI 1.00–1.41). In the chemotherapy-only subgroup (*n* = 767), no quintile differed significantly from the reference. Among patients who received no treatment (*n* = 448), only Q5 was associated with a significantly higher hazard of death (aHR 1.36, 95% CI 1.01–1.84). Exclusion of inferred-stage patients did not meaningfully alter the direction, magnitude, or statistical significance of the primary findings.

## 4. Discussion

In this population-based study of nearly 10,000 individuals with stage II-IV EOC treated within a universal healthcare system, distinct dimensions of marginalization exerted significant and divergent effects on survival. Material deprivation emerged as the strongest and most clinically meaningful determinant of worse survival, whereas residence in highly racialized or newcomer communities was contrastingly associated with improved survival. In the treatment stratified analysis of the Material Resources dimension, survival gradients were only identifiable across quintiles among individuals being treated with PCS or NACT, treatments that involve multimodal, multi-visit care over extended time periods. These population-level findings provide evidence that multidimensional social marginalization influence the survival of patients with EOC in Canada, further challenging the assumptions that universal health insurance alone may combat inequities in high-mortality cancers [22,23,24].

The association between material deprivation and EOC survival is congruent with previous studies that looked at similar exposures [14,25,26,27,28,29]. However, this is particularly notable within the Canadian context, as direct financial barriers to access healthcare do not exist, unlike other healthcare systems where insurance status or financial barriers more clearly drive disparities in access. These findings suggest that there are additional structural barriers that may be influencing outcomes, unrelated to direct healthcare costs. Postulated mechanisms related to material deprivation have included competing life demands (e.g., unstable employment, caregiving challenges, financial instability) leading to delayed symptom recognition or hindered healthcare seeking behaviour, limited access to transportation that deter frequent appointments, and chronic physiologic stress associated with long-term socioeconomic adversity [30,31,32]. The consistent, dose-dependent pattern observed in the adjusted model for material deprivation supports a structural, rather than behavioural, explanation for these disparities.

The observed protective association in neighbourhoods with higher proportions of racialized and newcomer communities conflicts with many findings reported from the US, where racialized groups often experience inferior outcomes related to delayed diagnosis and undertreatment [14,25,26,28,33,34,35,36]. This likely reflects a unique combination of contextual and systemic factors within Canada. Canada’s points-based immigration system selects for individuals with higher baseline health status, contributing to the well-documented Healthy Immigrant Effect [37]. It has been described that recent immigrants to Canada are significantly less likely to be diagnosed with any cancer [37]. Racialized and newcomer communities in Ontario are predominantly urban and often located near specialized cancer centres, where access to centralized gynecologic oncology expertise is known to improve survival [18]. Unmeasured community-level supports, including strong family networks or culture norms that promote healthcare engagement, may also play a role [38]. These results should be interpreted with the important caveat that ON-Marg is an area-level measure, where the observed associations reflect neighbourhood-level composition and should not be interpreted as individual-level race, ethnicity, or immigration effects. Our findings however signal that racialization and immigration are not inherently markers of vulnerability, and rather, its implications depend on social context. This is limited by the fact that not all Ontarian immigrants are captured in our cohort, including refugees (federally funded) and non-status migrants, however these are special populations with distinct health needs.

The treatment-stratified findings clarify how marginalization intersects with the cancer care continuum and challenge prior literature suggesting that survival disparities disappear when care is equivalent [39]. The median OS of 70.7 months in the PCS subgroup exceeds that reported in most randomized controlled trials (RCT) comparing PCS to NACT. This can be explained by several factors: our PCS cohort included stage II patients (13.7%, who have substantially better prognosis and are typically excluded from RCTs) and patients with algorithmically inferred advanced stage (37.6%, some of whom may have lower disease burden than RCT populations). The nearly twofold difference in median OS between PCS and NACT groups reflects some selection bias inherent to treatment assignment in real-world data, as patients selected for PCS tend to have favourable clinical characteristics, while patients triaged to NACT typically have higher disease burden and greater comorbidity. Importantly, our treatment-stratified analyses were designed to evaluate associations between marginalization and OS within treatment strata, adjusted for stage, rather than to compare survival across treatment groups. Acknowledging this, survival gradients were only identifiable across Material Resources quintiles among individuals being treated with PCS or NACT, treatments that generally involve multimodal, multi-visit care over extended time periods. Patients with limited material resources may face disproportionate challenges in navigating complex pathways. Longer survival amongst patients undergoing PCS or NACT provides a greater window of time for social inequities and material deprivation to influence long-term outcomes. In contrast, the profoundly limited survival among patients receiving chemotherapy only (12.4 months) or no treatment (2.3 months) may constrain the time in which social determinants can exert measurable effects, resulting in no survival differences amongst quintiles once restricted to these treatment subgroups. Clinical severity and limited survival may overshadow any measurable influence of social marginalization in these groups.

It should be noted that treatment received after diagnosis is not a simple baseline covariate and may be influenced by multiple factors such as disease severity, frailty, access to gynecologic oncology care. Treatment may lie on the causal pathway between marginalization and survival (e.g., material deprivation could affect access to timely surgery or chemotherapy completion, affecting survival). We adjusted for treatment in the main model, as the primary objective of this study was to estimate the direct effect of marginalization on survival independent of the treatment received. It is noted that adjusting for treatment in the main model may therefore block a pathway through which marginalization affects survival, potentially underestimating the total effect of marginalization on OS. A formal mediation analysis examining the extent to which treatment mediates the relationship between marginalization and survival would be an important direction for future work.

This work adds to the existing discourse on marginalization in cancer care as the only contemporaneous population-based analysis of Canadian EOC patients to date. As a population-based cohort study, there are limitations to acknowledge. ON-Marg is an area-level measure and does not capture individual socioeconomic characteristics; misclassification bias exists as important SDH may differ between individuals within the same neighbourhood, and thus it is not possible to elucidate causal relationships [40]. Further, although ON-Marg is multidimensional, it does not address all forms of marginalization experienced by all oppressed communities (e.g., indigenous populations, LGBTQ+ populations). The administrative data does not include individuals with no provincial insurance (such as those with federally funded healthcare), which comprises some of the most structurally marginalized populations (e.g., incarcerated individuals, some indigenous people, people who are homeless, refugee claimants, or non-status migrants), likely underestimating inequities in our study. The four ON-Marg dimensions may be correlated, as communities that experience heightened material deprivation, housing instability, and racialized/newcomer composition may overall geographically. An approach using four separate models was chosen a priori to identify the dimension most strongly associated with survival. However, this approach does not demonstrate whether each domain has an independent association with survival after accounting for the others and should not be interpreted as such. Lastly, stage was algorithmically inferred for a subset of patients, at potential risk for misclassification bias. However, the aHR in the material deprivation model for this ‘unknown/advanced’ subgroup (aHR 2.34, 95% CI 2.06–2.66) was comparable to, though slightly lower than, known stage III (aHR 2.52, 95% CI 2.23–2.86), suggesting this subgroup may include some patients with lower disease burden. Any bias results towards the null, rather than inflate the observed associations between marginalization and survival. Sensitivity analyses excluding all inferred-stage patients confirmed that the direction, magnitude, and statistical significance of the primary findings were preserved, further supporting that inclusion of this subgroup did not materially bias the conclusions of the study. Additional, treatment groups were defined after diagnosis, introducing the possibility of immortal time bias; however, as the treatment-stratified analyses evaluate associations between marginalization and OS within treatment groups, rather than comparing survival between them, this is unlikely to meaningfully influence our primary findings.

## 5. Conclusions

Material deprivation emerged as the strongest independent predictor of survival in EOC, even within a universal healthcare system, with the greatest impact observed among patients undergoing complex, multimodal treatment pathways. Conversely, residence in highly racialized or newcomer communities was associated with improved survival, highlighting that different social dimensions of marginalization exert distinct and context-dependent effects. This should be carefully interpreted within the context of a universal healthcare system and a cohort limited to individuals eligible for publicly funded care. These findings underscore the need for targeted, system-level interventions that improve care coordination and address material barriers to engaging with complex oncologic care, supporting equitable access to optimal ovarian cancer treatment across all communities. Efforts to improve equity should focus on health system interventions that address financial, geographic, and logistical barriers that may limit access to specialized oncologic care. Future work should investigate the patient- and system-level mechanisms underlying these survival disparities, and evaluate targeted interventions designed to mitigate the impact of marginalization on cancer outcomes.

## Figures and Tables

**Table 1:** Cohort characteristics per treatment group.

Characteristics	Overall Cohort	Treatment Subgroups				
		PCS *	NACT **	Chemo Only	No Treatment	*p*-Value
*N* (%)	9613	5189 (54.0)	2567 (26.7)	1409 (14.7)	448 (4.7)	
Age at Dx, y—mean (SD)	64.00 (12.96)	60.69 (13.10)	64.47 (10.32)	71.57 (11.35)	75.79 (12.31)	<0.0001
Year at Dx—*n* (%)						
2010–2012	2104 (21.9)	1036 (20.0)	547 (21.3)	313 (22.2)	208 (46.4)	<0.0001
2013–2015	2110 (21.9)	1214 (23.4)	492 (19.2)	316 (22.4)	88 (19.6)	
2016–2018	2282 (23.7)	1246 (24.0)	644 (25.1)	312 (22.1)	80 (17.9)	
2019–2022	3117 (32.4)	1693 (32.6)	884 (34.4)	468 (33.2)	72 (16.1)	
Cancer Stage at Dx—*n* (%)						
II	783 (8.1)	713 (13.7)	43 (1.7)	10 (0.7)	17 (3.8)	<0.0001
III	3671 (38.2)	2049 (39.5)	1083 (42.2)	346 (24.6)	193 (43.1)	
IV	1705 (17.7)	474 (9.1)	582 (22.7)	411 (29.2)	238 (53.1)	
Unknown, Advanced	3454 (35.9)	1953 (37.6)	859 (33.5)	642 (45.6)	0 (0.0)	
Elixhauser Comorbidity Index—*n* (%)						
0–3	9220 (95.9)	5015 (96.6)	2500 (97.4)	1301 (92.3)	404 (90.2)	<0.0001
4+	393 (4.1)	174 (3.4)	67 (2.6)	108 (7.7)	44 (9.8)	
ON-Marg: Material Resources—*n* (%)						
Q1 (least marginalized)	2008 (20.9)	1099 (21.2)	558 (21.7)	272 (19.3)	79 (17.6)	<0.0001
Q2	2063 (21.5)	1153 (22.2)	548 (21.3)	279 (19.8)	83 (18.5)	
Q3	1922 (20.0)	1007 (19.4)	547 (21.3)	291 (20.7)	77 (17.2)	
Q4	1903 (19.8)	1043 (20.1)	467 (18.2)	303 (21.5)	90 (20.1)	
Q5 (most marginalized)	1717 (17.9)	887 (17.1)	447 (17.4)	264 (18.7)	119 (26.6)	
ON-Marg: Households and Dwellings—*n* (%)						
Q1 (least marginalized)	1754 (18.2)	1047 (20.2)	451 (17.6)	212 (15.0)	44 (9.8)	<0.0001
Q2	1823 (19.0)	983 (18.9)	514 (20.0)	269 (19.1)	57 (12.7)	
Q3	1788 (18.6)	930 (17.9)	484 (18.9)	280 (19.9)	94 (21.0)	
Q4	1938 (20.2)	1025 (19.8)	516 (20.1)	293 (20.8)	104 (23.2)	
Q5 (most marginalized)	2310 (24.0)	1204 (23.2)	602 (23.5)	355 (25.2)	149 (33.3)	
ON-Marg: Age and Labour Force—*n* (%)						
Q1 (least marginalized)	1790 (18.6)	1064 (20.5)	473 (18.4)	195 (13.8)	58 (12.9)	<0.0001
Q2	1792 (18.6)	1001 (19.3)	491 (19.1)	225 (16.0)	75 (16.7)	
Q3	1702 (17.7)	931 (17.9)	459 (17.9)	251 (17.8)	61 (13.6)	
Q4	1827 (19.0)	939 (18.1)	487 (19.0)	308 (21.9)	93 (20.8)	
Q5 (most marginalized)	2502 (26.0)	1254 (24.2)	657 (25.6)	430 (30.5)	161 (35.9)	
ON-Marg: Racialized and Newcomer Populations—*n* (%)						
Q1 (least marginalized)	1805 (18.8)	902 (17.4)	507 (19.8)	303 (21.5)	93 (20.8)	0.0007
Q2	1820 (18.9)	954 (18.4)	489 (19.0)	279 (19.8)	98 (21.9)	
Q3	1799 (18.7)	967 (18.6)	491 (19.1)	259 (18.4)	82 (18.3)	
Q4	1967 (20.5)	1075 (20.7)	524 (20.4)	280 (19.9)	88 (19.6)	
Q5 (most marginalized)	2222 (23.1)	1291 (24.9)	556 (21.7)	288 (20.4)	87 (19.4)	

* PCS—Primary Cytoreductive Surgery; ** NACT—Neoadjuvant Chemotherapy with Interval Debulking Surgery.

**Table 2:** Multivariable Cox proportional hazards models examining overall survival across four different ON-Marg dimensions of marginalization, each substituted into otherwise identical models (*n* = 9613).

	MODEL: Material Resources		MODEL: Households and Dwellings		MODEL: Age and Labour Force		MODEL: Racialized and Newcomer Pop.	
	HR (95% CI)	*p*-Value	HR (95% CI)	*p*-Value	HR (95% CI)	*p*-Value	HR (95% CI)	*p*-Value
Age at Diagnosis	1.03 (1.02–1.03)	<0.001	1.03 (1.02–1.03)	<0.001	1.03 (1.02–1.03)	<0.001	1.03 (1.02–1.03)	<0.001
Year of Diagnosis	0.97 (0.96–0.98)	<0.001	0.97 (0.96–0.98)	<0.001	0.97 (0.96–0.98)	<0.001	0.97 (0.96–0.98)	<0.001
Stage at Diagnosis								
Stage II	Reference		Reference		Reference		Reference	
Stage III	2.52 (2.23–2.86)	<0.001	2.53 (2.24–2.87)	<0.001	2.53 (2.23–2.86)	<0.001	2.53 (2.23–2.86)	<0.001
Stage IV	3.04 (2.66–3.47)	<0.001	3.06 (2.68–3.50)	<0.001	3.06 (2.68–3.49)	<0.001	3.05 (2.67–3.49)	<0.001
Unknown, Advanced	2.34 (2.06–2.66)	<0.001	2.36 (2.08–2.68)	<0.001	2.34 (2.06–2.66)	<0.001	2.36 (2.08–2.68)	<0.001
Elixhauser Comorbidity Index								
0–3	Reference		Reference		Reference		Reference	
4+	1.36 (1.21–1.52)	<0.001	1.37 (1.22–1.53)	<0.001	1.36 (1.22–1.53)	<0.001	1.36 (1.22–1.53)	<0.001
Treatment Received								
Primary cytoreductive surgery	Reference		Reference		Reference		Reference	
Neoadjuvant chemotherapy	1.42 (1.34–1.51)	<0.001	1.42 (1.33–1.51)	<0.001	1.42 (1.34–1.51)	<0.001	1.42 (1.34–1.51)	<0.001
Chemotherapy only	3.04 (2.82–3.27)	<0.001	3.04 (2.82–3.27)	<0.001	3.04 (2.83–3.27)	<0.001	3.04 (2.83–3.28)	<0.001
No treatment	6.55 (5.87–7.31)	<0.001	6.58 (5.90–7.34)	<0.001	6.59 (5.91–7.35)	<0.001	6.59 (5.91–7.36)	<0.001
ON-Marg Dimension *—*n* (%)								
Q1 (least marginalized)	Reference		Reference		Reference		Reference	
Q2	1.03 (0.95–1.11)	0.47	1.09 (1.01–1.19)	0.03	1.04 (0.96–1.13)	0.32	1.01 (0.94–1.09)	0.78
Q3	1.10 (1.02–1.19)	0.02	1.09 (1.00–1.18)	0.05	1.05 (0.97–1.15)	0.23	0.97 (0.89–1.05)	0.40
Q4	1.13 (1.05–1.22)	0.001	1.13 (1.04–1.22)	0.004	1.09 (1.00–1.18)	0.05	0.91 (0.84–0.98)	0.01
Q5 (most marginalized)	1.25 (1.15–1.35)	<0.001	1.12 (1.03–1.21)	0.01	1.04 (0.96–1.13)	0.31	0.87 (0.80–0.94)	<0.001

* ON-Marg Dimension specific to each model.

## Data Availability

The dataset from this study is held securely in coded form at ICES. While data-sharing agreements prohibit ICES from making the dataset publicly available, access may be granted to those who meet prespecified criteria for confidential access, available at www.ices.on.ca/DAS (accessed on 1 May 2026).

## Supplementary Materials

The following supporting information can be downloaded at: https://www.mdpi.com/article/10.3390/cancers18121892/s1, Supplementary Table S1. Cohort characteristics stage at presentation; Supplementary Table S2. Sensitivity Analysis—Multivariable Cox proportional hazards models examining overall survival across four different ON-Marg dimensions of marginalization, each substituted into otherwise identical models, only including patients with known stage (*n* = 6159); Supplementary Table S3. Sensitivity Analysis—Treatment stratified multivariable Cox proportional hazards models examining overall survival for ON-Marg Material Resources dimensions, only including patients with known stage.
