# Supplementary material for: Impact of Marginalization Dimensions on Survival Disparities in Epithelial Ovarian Cancer: An Ontario Population-Based Study
Source: Cancers (Basel). 2026 Jun 10;18(12):1892. doi: 10.3390/cancers18121892 (PMC13296536; doi:10.3390/cancers18121892)
Supplement: Supplementary file 1 [file cancers-18-01892-s001.zip › cancers-4289730-supplementary.pdf]

Supplementary Table 1. Cohort characteristics stage at presentation

Characteristics	Overall Cohort	Stage				
		Known Stage II	Known Stage III	Known Stage IV	Unknown, Advanced	p-value
N	9,613	783	3671	1705	3454	
Age at Dx, years – mean (SD)	64.00 (12.96)	61.05 (12.34)	64.18 (12.31)	66.52 (12.66)	63.22 (13.67)	<0.0001
Year at Dx – n (%)						<0.0001
2010 – 2012	2,104 (21.9)	230 (29.4)	1,081 (29.4)	443 (26.0)	350 (10.1)	
2013 – 2015	2,110 (21.9)	206 (26.3)	916 (25.0)	349 (20.5)	639 (18.5)	
2016 – 2018	2,282 (23.7)	169 (21.6)	787 (21.4)	393 (23.0)	933 (27.0)	
2019 – 2022	3,117 (32.4)	178 (22.7)	887 (24.2)	520 (30.5)	1,532 (44.4)	
Elixhauser Comorbidity Index – n (%)						0.00118
0 – 3	9,220 (95.9)	754 (96.3)	3,536 (96.3)	1,611 (94.5)	3,319 (96.1)	
4+	393 (4.1)	29 (3.7)	135 (3.7)	94 (5.5)	135 (3.9)	
ON-Marg: Material Resources – n (%)						0.0069
Q1 *(least marginalized)*	2,008 (20.9)	179 (22.9)	786 (21.4)	350 (20.5)	693 (20.1)	
Q2	2,063 (21.5)	165 (21.1)	794 (21.6)	363 (21.3)	741 (21.5)	
Q3	1,922 (20.0)	148 (18.9)	702 (19.1)	326 (19.1)	746 (21.6)	
Q4	1,903 (19.8)	161 (20.6)	724 (19.7)	310 (18.2)	708 (20.5)	
Q5 *(most marginalized)*	1,717 (17.9)	130 (16.6)	665 (18.1)	356 (20.9)	566 (16.4)	
ON-Marg: Households and Dwellings – n (%)						<0.0001
Q1 *(least marginalized)*	1,754 (18.2)	137 (17.5)	609 (16.6)	274 (16.1)	734 (21.3)	
Q2	1,823 (19.0)	139 (17.8)	700 (19.1)	314 (18.4)	670 (19.4)	
Q3	1,788 (18.6)	145 (18.5)	696 (19.0)	300 (17.6)	647 (18.7)	
Q4	1,938 (20.2)	172 (22.0)	754 (20.5)	350 (20.5)	662 (19.2)	
Q5 *(most marginalized)*	2,310 (24.0)	190 (24.3)	912 (24.8)	467 (27.4)	741 (21.5)	
ON-Marg: Age and Labour Force – n (%)						0.0086
Q1 *(least marginalized)*	1,790 (18.6)	145 (18.5)	645 (17.6)	297 (17.4)	703 (20.4)	
Q2	1,792 (18.6)	140 (17.9)	714 (19.4)	317 (18.6)	621 (18.0)	
Q3	1,702 (17.7)	157 (20.1)	638 (17.4)	276 (16.2)	631 (18.3)	
Q4	1,827 (19.0)	137 (17.5)	688 (18.7)	338 (19.8)	664 (19.2)	
Q5 *(most marginalized)*	2,502 (26.0)	204 (26.1)	986 (26.9)	477 (28.0)	835 (24.2)	
ON-Marg: Racialized and Newcomer Populations – n (%)						<0.0001
Q1 *(least marginalized)*	1,805 (18.8)	143 (18.3)	699 (19.0)	336 (19.7)	627 (18.2)	
Q2	1,820 (18.9)	146 (18.6)	737 (20.1)	346 (20.3)	591 (17.1)	
Q3	1,799 (18.7)	172 (22.0)	718 (19.6)	316 (18.5)	593 (17.2)	
Q4	1,967 (20.5)	166 (21.2)	755 (20.6)	350 (20.5)	696 (20.2)	
Q5 *(most marginalized)*	2,222 (23.1)	156 (19.9)	762 (20.8)	357 (20.9)	947 (27.4)	
Treatment						<0.0001
PCS	5,189 (54.0)	713 (91.1)	2,049 (55.8)	474 (27.8)	1,953 (56.5)	
NACT	2,567 (26.7)	43 (5.5)	1,083 (29.5)	582 (34.1)	859 (24.9)	
Chemo	1,409 (14.7)	10 (1.3)	346 (9.4)	411 (24.1)	642 (18.6)	
None	448 (4.7)	17 (2.2)	193 (5.3)	238 (14.0)	0 (0.0)	

*PCS – Primary Cytoreductive Surgery
**NACT – Neoadjuvant Chemotherapy with Interval Debulking Surgery

Supplementary Table 2. Sensitivity Analysis - Multivariable Cox proportional hazards models examining overall survival across four different ON-Marg dimensions of marginalization, each substituted into otherwise identical models, only including patients with known stage (n = 6,159)

	MODEL: Material Resources		MODEL: Households & Dwellings		MODEL: Age & Labour Force		MODEL: Racialized & Newcomer Pop.	
	HR (95% CI)	p-value	HR (95% CI)	p-value	HR (95% CI)	p-value	HR (95% CI)	p-value
Age at Diagnosis	1.02 (1.02-1.03)	<0.001	1.02 (1.02-1.03)	<0.001	1.02 (1.02-1.03)	<0.001	1.02 (1.02-1.03)	<0.001
Year of Diagnosis	0.97 (0.96-0.98)	<0.001	0.97 (0.96-0.98)	<0.001	0.97 (0.96-0.98)	<0.001	0.97 (0.96-0.98)	<0.001
Stage at Diagnosis								
Stage II	Reference		Reference		Reference		Reference	
Stage III	2.72 (2.40-3.08)	<0.001	2.73 (2.41-3.09)	<0.001	2.72 (2.40-3.09)	<0.001	2.72 (2.40-3.08)	<0.001
Stage IV	3.47 (3.03-3.97)	<0.001	3.49 (3.05-4.00)	<0.001	3.49 (3.04-3.99)	<0.001	3.47 (3.03-3.98)	<0.001
Elixhauser Comorbidity Index								
0 – 3	Reference		Reference		Reference		Reference	
4+	1.45 (1.26-1.67)	<0.001	1.47 (1.28-1.69)	<0.001	1.46 (1.27-1.68)	<0.001	1.46 (1.28-1.68)	<0.001
Treatment Received								
Primary cytoreductive surgery	Reference		Reference		Reference		Reference	
Neoadjuvant chemotherapy	1.25 (1.16-1.35)	<0.001	1.25 (1.16-1.35)	<0.001	1.26 (1.17-1.35)	<0.001	1.26 (1.17-1.35)	<0.001
Chemotherapy only	2.52 (2.29-2.78)	<0.001	2.52 (2.29-2.78)	<0.001	2.53 (2.30-2.78)	<0.001	2.53 (2.29-2.78)	<0.001
No treatment	6.26 (5.59-7.01)	<0.001	6.30 (5.63-7.05)	<0.001	6.31 (5.64-7.06)	<0.001	6.31 (5.63-7.06)	<0.001
ON-Marg Dimension* – n (%)								
Q1 *(least marginalized)*	Reference		Reference		Reference		Reference	
Q2	1.01 (0.92-1.11)	0.8616	1.12 (1.01-1.24)	0.0361	1.02 (0.92-1.13)	0.6559	1.00 (0.91-1.10)	0.9895
Q3	1.08 (0.98-1.18)	0.1212	1.10 (0.99-1.22)	0.0639	1.06 (0.96-1.18)	0.2678	0.92 (0.84-1.01)	0.0901
Q4	1.11 (1.01-1.22)	0.0278	1.11 (1.00-1.23)	0.0402	1.09 (0.99-1.21)	0.0856	0.89 (0.81-0.98)	0.0151
Q5 *(most marginalized)*	1.21 (1.10-1.33)	<.0001	1.14 (1.03-1.25)	0.0093	1.04 (0.95-1.15)	0.3747	0.89 (0.81-0.98)	0.015

*ON-Marg Dimension specific to each model

Supplementary Table 3. Sensitivity Analysis - Treatment stratified multivariable Cox proportional hazards models examining overall survival for ON-Marg Material Resources dimensions, only including patients with known stage

	PCS n = 3,236		NACT n = 1,708		Chemo Only: n = 767		No Treatment n = 448	
	HR (95% CI)	p-value	HR (95% CI)	p-value	HR (95% CI)	p-value	HR (95% CI)	p-value
Age at Diagnosis	1.02 (1.02-1.03)	<.0001	1.01 (1.01-1.02)	<.0001	1.01 (1.00-1.01)	0.0916	1.02 (1.02-1.03)	<.0001
Year of Diagnosis	0.97 (0.96-0.99)	<.0001	0.98 (0.97-1.00)	0.0263	0.97 (0.95-0.98)	0.0004	0.97 (0.94-0.99)	0.0138
Stage at Diagnosis								
Stage II	Reference		Reference		Reference		Reference	
Stage III	2.86 (2.49-3.29)	<.0001	1.59 (1.09-2.31)	0.0149	1.82 (0.80-4.15)	0.1532	1.75 (1.01-3.05)	0.0463
Stage IV	4.15 (3.50-4.91)	<.0001	1.71 (1.17-2.50)	0.0057	2.20 (0.97-5.01)	0.0589	2.32 (1.34-4.03)	0.0027
Elixhauser Comorbidity Index								
0 – 3	Reference		Reference		Reference		Reference	
4+	1.61 (1.28-2.03)	<.0001	1.26 (0.91-1.74)	0.1587	1.23 (0.94-1.62)	0.1272	1.33 (0.97-1.83)	0.0771
ON-Marg Dimension* – n (%)								
Q1 *(least marginalized)*	Reference		Reference		Reference		Reference	
Q2	0.92 (0.81-1.06)	0.2629	1.05 (0.89-1.24)	0.5683	0.91 (0.71-1.15)	0.4221	1.30 (0.94-1.79)	0.1188
Q3	1.01 (0.88-1.16)	0.8905	1.09 (0.92-1.28)	0.334	1.00 (0.79-1.27)	0.9922	1.25 (0.90-1.74)	0.1861
Q4	1.07 (0.93-1.23)	0.3258	1.18 (1.00-1.40)	0.0551	1.00 (0.79-1.25)	0.9733	1.30 (0.94-1.79)	0.1093
Q5 *(most marginalized)*	1.16 (1.01-1.34)	0.0379	1.19 (1.00-1.41)	0.0485	1.12 (0.88-1.42)	0.3522	1.36 (1.01-1.84)	0.0441

*PCS – Primary Cytoreductive Surgery
**NACT – Neoadjuvant Chemotherapy with Interval Debulking Surgery
